# The Influence of Social Rejection on Non-Suicidal Self-Injury Among Chinese Adolescents: A Longitudinal Moderated Mediation Model

**DOI:** 10.3390/bs16081281

**Published:** 2026-07-27

**Authors:** Shuang Lin, Jiamin Liang, Yongjian Li, Jun Chen, Huifang Yang, Yulin Li, Yuwen Lin, Lijun Chen

**Affiliations:** 1 School of Educational Science, Guangdong Polytechnic Normal University, Guangzhou 510665, China; linshuang@gpnu.edu.cn (S.L.);; 2 School of Psychology, South China Normal University, 55 West Zhongshan Road, Guangzhou 510631, China

**Keywords:** non-suicidal self-injury, social rejection, self-esteem, anxiety and depression, longitudinal study

## Abstract

Social rejection is a risk factor for non-suicidal self-injury (NSSI) among adolescents. However, little is known about the longitudinal mediating and moderating mechanisms underlying the relationship between social rejection and NSSI. This study aimed to investigate the mediating role of anxiety and depression symptoms between social rejection and NSSI, as well as the moderating role of self-esteem among adolescents. A total of 1368 Chinese middle school students (60% boys, *n* = 821; 40% girls, *n* = 547; *M_age_* = 16.05 years, *SD* = 0.85 at T1) participated in the study. They completed measures of social rejection, NSSI, anxiety and depression symptoms, and self-esteem over three waves (T1, T2, and T3), each six months apart. Longitudinal mediation models indicated that T1 social rejection is linked to T3 NSSI by predicting higher levels of T2 anxiety and depression symptoms. Additionally, T2 self-esteem moderates this indirect effect; specifically, the association between social rejection and anxiety and depression is significant only in adolescents with low self-esteem. These findings underscore the importance of addressing anxiety and depression symptoms and enhancing adolescent self-esteem in the prevention and intervention of NSSI.

## 1. Introduction

Non-suicidal self-injury (NSSI) is defined as “the direct and intentional destruction of one’s own body such as self-cutting or self-burning, but without the intent to die” (Nock, 2010). Recent studies have shown that NSSI is a major public mental health problem among adolescents; as many as 16% to 29% of Chinese adolescents report lifetime histories of NSSI (Lin et al., 2025; Zhou & Gong, 2023). NSSI has received increasing attention from researchers because of its association with various psychiatric disorders, such as borderline personality disorder and major depression (Cipriano et al., 2017), and even as a significant predictor of suicidal ideation and suicidal behavior (Kiekens et al., 2018). Given the high prevalence and serious consequences of NSSI among adolescents, a better understanding of the underlying factors associated with the development and maintenance of NSSI is imperative for the development of new intervention and prevention strategies.

Adolescence is a developmental period marked by heightened social evaluative concerns, such as increased attention to the perceptions of parents, peers, and friends, as well as greater preoccupation with one’s own social performance in social interactions (e.g., Masten et al., 2009; Westenberg et al., 2007). Recent meta-analytic evidence indicated an association between experiences of social rejection and engagement in self-injurious behavior among adolescents (Cheek et al., 2020). However, the mechanisms and conditions underlying the association between social rejection and NSSI remain unclear. The current study aimed to address this research gap by examining the longitudinal relationship between experiences of social rejection and engagement in NSSI among Chinese adolescents over time, controlling for prior NSSI (assessed at T2). Additionally, this study explored the effect of anxiety and depression as well as self-esteem on the relationship between social rejection and NSSI.

### 1.1. Social Rejection and NSSI

The integrated theoretical model of NSSI proposed by Nock (2010) provides a comprehensive framework for understanding the development of self-injurious behaviors. According to this model, negative events elicit intense negative emotions, which individuals may then seek to regulate through engagement in NSSI as a maladaptive coping strategy. Supporting this notion, the most commonly reported reason for self-injurious behavior is the desire to alleviate distressing feelings (Nock & Prinstein, 2004). Within this theoretical framework, social rejection can be conceptualized as a prototypical negative event, a distressing experience of being intentionally excluded from desired groups, relationships, or interactions (Bierman, 2004; Ophir, 2017). Experiences of social rejection can elicit various cognitive–emotional responses, including anxiety, self-blame, emotional distress, and hostility (Jiang et al., 2020). In line with Nock‘s model, these responses represent the intense negative emotions that arise from the negative event. Specifically, anxiety and depression can be understood as the internalizing emotional states that emerge following social rejection, which individuals may be motivated to regulate or escape from. To alleviate these aversive emotional states, individuals may resort to NSSI as a maladaptive emotion regulation strategy. In addition, self-esteem may moderate this pathway, such that adolescents with lower self-esteem are more susceptible to the emotional consequences of rejection and thus at greater risk for NSSI. Under the framework of the integrated developmental model of NSSI, the current study focused on the pathway from social rejection to NSSI, examining the mediating role of anxiety and depression, and investigating whether self-esteem moderates this process. To the best of our knowledge, few longitudinal studies have examined these relationships simultaneously.

Previous studies have shown a direct correlation between social rejection and NSSI. A cross-sectional study of 728 Chinese adolescents demonstrated a significant positive correlation between experiences of rejection and engagement in NSSI (Jiang et al., 2020). Furthermore, a meta-analysis of 56 studies found a consistent association between experiences of social rejection, such as low relationship evaluation, and engagement in self-injurious behaviors (Cheek et al., 2020). In addition, a neuroimaging study demonstrated that adolescents with NSSI exhibit heightened activation in the medial prefrontal cortex (mPFC) and ventrolateral prefrontal cortex (vlPFC) following induced feelings of social rejection compared to healthy controls (Groschwitz et al., 2016). However, most prior research has relied on cross-sectional designs, which preclude conclusions about directionality and temporal relationships. Therefore, the current study utilized a one-year longitudinal design while controlling for prior NSSI (assessed at T2) to examine the relationship between social rejection and NSSI over time, as well as potential underlying mechanisms.

### 1.2. The Mediating Role of Anxiety and Depression

The relationship between social rejection and engagement in NSSI may be mediated by symptoms of anxiety and depression. On the one hand, symptoms of anxiety and depression are implicated in the development and maintenance of NSSI (e.g., Marshall et al., 2013; Zhu et al., 2021). The affect regulation model proposes that NSSI represents a maladaptive strategy to alleviate emotional distress among adolescents (Klonsky, 2007). Adolescents experiencing negative emotions may engage in self-harming behaviors like NSSI, as these can rapidly and effectively alleviate dysphoric moods (Nock, 2010). A substantial body of research indicates that symptoms of anxiety and depression can prospectively predict engagement in NSSI (Plener et al., 2015; Valencia-Agudo et al., 2018; Zhu et al., 2021).

On the other hand, social rejection may increase the risk of anxiety and depression. Adolescents are sensitive to interpersonal relationships and may experience strong emotional reactions, including stress, anxiety, anger, rumination, and helplessness, in response to social rejection (Jiang et al., 2020). Maladaptive behavioral responses, like social withdrawal or aggression, can further contribute to social isolation and loneliness. The cumulative interaction between interpersonal stressors and psychological distress may manifest in symptoms of anxiety and depression (Cheek et al., 2020; Zhu et al., 2021). Despite prior research indicating that anxiety and depression are associated with social rejection and NSSI, there is limited exploration into how anxiety and depression mediate the relationship between social rejection and NSSI. A comprehensive theoretical model of NSSI (Nock, 2010) provides a framework for understanding how anxiety and depression mediate the link between social rejection and NSSI. Social rejection can elevate anxiety and depression. To alleviate these emotional states, individuals may engage in NSSI. Longitudinal studies have provided some evidence that anxiety and depression mediated the relationship between adverse stressful experiences and NSSI (Li et al., 2023a; Zhu et al., 2021). Although anxiety and depression are conceptually distinct dimensions, they exhibit substantial comorbidity in adolescent populations, with estimated rates ranging from 15.9% to 61.9% (Cummings et al., 2014), and share significant conceptual overlap as broad internalizing distress (Choi et al., 2020). Factor analytic studies have supported a single-factor model of anxiety and depressive disorders in adolescent samples, suggesting that they can be parsimoniously examined as a combined internalizing construct (Hale et al., 2009). Given this empirical and theoretical support, we modeled anxiety and depression as a single composite construct of internalizing symptoms in the present study. Drawing from both theoretical frameworks and empirical evidence, we employed a one-year longitudinal study to investigate whether anxiety and depression mediate the relationship between social rejection and NSSI.

### 1.3. The Moderating Role of Self-Esteem

Although social rejection is a risk factor for NSSI, not all adolescents who experience social rejection would engage in NSSI. Self-esteem is one of the personal characteristics that may help buffer the relationship between social rejection and NSSI. Self-esteem refers to an individual’s positive self-evaluation or their assessment of their own worth (Leary & Baumeister, 2000). Individuals exhibiting high self-esteem demonstrate enhanced clarity and confidence (Heimpel et al., 2002), equipping them with additional resources to navigate stressful situations and decreasing the likelihood of engaging in negative coping or in risky behaviors (Cieślak & Golusiński, 2018; West, 2018). In contrast, low self-esteem entails a more pessimistic self-assessment and self-perception, predisposing individuals to adopt negative coping strategies and engage in risky behaviors. To understand why self-esteem functions as a protective factor in this context, we draw on the Conservation of Resources (COR) theory (Hobfoll, 1989). Nock’s (2010) integrated model explains the general mechanism by which negative events lead to NSSI through the generation of intense negative emotions that individuals seek to regulate. COR theory provides a complementary framework for understanding individual differences in this process. According to COR theory, individuals strive to obtain, retain, and protect their personal resources (e.g., self-esteem, social support, coping abilities). When these resources are threatened or lost, stress ensues, and individuals may adopt maladaptive coping strategies to manage resource depletion. Within this framework, self-esteem can be understood as a key personal resource that buffers the impact of stressful events on maladaptive behaviors. Specifically, adolescents with high self-esteem possess greater psychological resources to cope with the distress of social rejection, whereas those with low self-esteem lack this resource, making them more vulnerable to emotional distress and subsequent NSSI. Thus, COR theory offers a theoretically grounded explanation for why self-esteem moderates the pathway from social rejection to NSSI via anxiety and depression.

Past studies have indicated that self-esteem moderated the association between adverse stressful events and maladaptive behaviors (Gao et al., 2024; Zheng et al., 2020). For instance, a recent two-wave longitudinal study indicated that self-esteem may serve as a protective factor, mitigating the relationship between recent stressful events and NSSI among adolescents exposed to child maltreatment (Gao et al., 2024). More directly relevant to the present study, studies have found that self-esteem moderates the relationship between social anxiety and self-injury, suggesting that individuals with lower self-esteem are more susceptible to the maladaptive consequences of social stressors (Zou et al., 2024). Individuals with varying levels of self-esteem exhibit distinct attitudes in self-evaluation. Those with low self-esteem frequently experience negative emotions, perceiving themselves as inadequate and less valuable compared to others (Lin et al., 2023). The literature indicated a negative correlation between self-esteem and depression (Orth & Robins, 2013; Zhou et al., 2020). Research indicates that individuals with high self-esteem are less susceptible to depression, while those with low self-esteem are more likely to experience depression when faced with negative stressful events (Varghese & Pistole, 2017). Individuals with high self-esteem tend to be more satisfied with life, achieve higher and more consistent levels of success, and have a lower risk of anxiety and depression compared to those with low self-esteem. High self-esteem may serve as a protective factor against anxiety and depression. Based on COR theory and empirical research, we employed a longitudinal design to examine whether self-esteem moderated the mediating effect of anxiety and depression on the relationship between social rejection and NSSI.

### 1.4. The Current Study

This study utilized a one-year longitudinal design to investigate how and when social rejection is associated with NSSI among adolescents. Based on the above theoretical framework and empirical research, we hypothesized that (1) T1 social rejection is linked to T3 NSSI by predicting higher levels of T2 anxiety and depression symptoms, and (2) T2 self-esteem moderates the mediating association between T1 social rejection and T3 NSSI.

## 2. Method

### 2.1. Participants

The sample of the current study was from a longitudinal study focusing on adolescents’ mental health (Lin et al., 2023). The analytic sample comprised 1368 adolescents recruited from four secondary schools in Guangdong Province. The data were collected in a one-year short-term longitudinal study, which was carried out at three points in time in six-month intervals. A total of 1655 adolescents participated at the initial assessment (T1). Of these, 287 participants (17.5%) were excluded from the final analytic sample due to attrition at T2 (9.5%) or T3 (17.5%), resulting in a final sample of 1368 adolescents with complete data across all three waves. The final sample consisted of 821 boys (60%) and 547 girls (40%), with a mean age of 16.05 years (SD = 0.85) at T1. Attrition analysis revealed no statistically significant differences between the final sample and the excluded cases on all variables. Family demographics showed 21.1% only children and 78.9% with siblings. Fathers’ educational attainment was 66.2% ≤ high school, 24.6% junior college, 6.3% bachelor’s, 3.0% graduate degrees, while for mothers it was 71.7%, 20.5%, 5.4%, and 2.4%, respectively. Perceived family economic status was predominantly moderate (82.0%), with other categories as very rich (0.4%), relatively well-off (4.2%), relatively poor (10.5%), and very poor (2.9%).

### 2.2. Measures

#### 2.2.1. Social Rejection

Social rejection was measured using four items (“feelings of loneliness”; “not getting along with peers”; “thinking that others want to hurt them” and “feeling that they are not loved”) from the social problems subscale of the Youth Self-Report (YSR; Achenbach, 1991). This measure has been widely employed in previous studies (e.g., Ophir, 2017; Ophir et al., 2019) and has shown satisfactory reliability and validity in Chinese adolescent samples (Li et al., 2023b). Participants rated each item on a 5-point scale ranging from 1 (*strongly disagree*) to 5 (*strongly agree*), indicating their perceived level of social rejection. In the current sample, internal consistency was high across all waves, with Cronbach’s α of 0.91 at T1, 0.94 at T2, and 0.95 at T3.

#### 2.2.2. Anxiety and Depression

Given their high comorbidity and shared conceptual overlap in adolescent populations (Choi et al., 2020; Cummings et al., 2014), anxiety and depression were modeled as a single composite construct of internalizing distress in the present study (Hale et al., 2009). At T1 and T2, this construct was measured using the Chinese version of the 16-item anxiety and depression scale (Yu et al., 2016). This instrument comprises 8 depression and 8 anxiety items, all rated on a 3-point Likert scale (1 = *never*, 3 = *often*). Higher mean scores indicated greater symptom severity. Good internal consistency was observed in the current sample across T1, T2, and T3, with Cronbach’s α values of 0.94, 0.95, and 0.96, respectively.

#### 2.2.3. Self-Esteem

At T2, we measured self-esteem using a self-report index of global self-esteem (Rosenberg, 1965). The scale contains 10 items (e.g., “I have a positive attitude toward myself”) and has demonstrated good reliability and validity in a sample of Chinese adolescents (Lin et al., 2023). All items were rated on a 4-point scale from 1 (*strongly disagree*) to 4 (*strongly agree*). The mean of the ten items was calculated, with higher scores indicating greater self-esteem. The measure yielded acceptable internal consistency in this sample at each time point (T1: α = 0.85; T2: α = 0.89; T3: α = 0.90).

#### 2.2.4. NSSI

A total of seven NSSI behaviors were assessed in the current study, including self-cutting, burning, biting, punching, scratching the skin, inserting objects into the nail or skin, and banging the head or other body parts against the wall. These items were selected because they are among the most common NSSI behaviors reported in adolescent populations (Nock, 2010) and have demonstrated satisfactory reliability and validity in previous studies with Chinese adolescents (Gong et al., 2019). Participants were asked whether they had engaged in any of these behaviors over the past 12 months, and responses were recorded on a 7-point Likert scale ranging from 1 (*never*) to 7 (*at least once per day*), with higher scores indicating greater NSSI frequency. In the current sample, the scale demonstrated excellent internal consistency, with Cronbach’s α values of 0.97 at both T2 and T3.

### 2.3. Procedure

The research was approved by the Ethics Committee of South China Normal University (No: SCNU-PSY-2022-325) and conducted in accordance with the ethical principles outlined in the Helsinki Declaration. Data collection commenced after obtaining informed consent from both participating students and their parents. Prior to data collection, participants were informed that their responses would be kept strictly confidential and could be withdrawn from the study at any time. Self-report questionnaires were administered by trained researchers.

### 2.4. Statistical Analysis

We used Mplus 8.0 to test the proposed longitudinal mediation model. For participants who completed all three waves, occasional item-level missingness was handled using full information maximum likelihood (FIML) estimation. The overall model fit was assessed by the chi-square to degrees of freedom ratio (χ^2^/df), comparative fit index (CFI), root mean square error of approximation (RMSEA), and standardized root mean square residual (SRMR), with acceptable fit criteria set at χ^2^/df < 5, CFI > 0.90, RMSEA < 0.08, and SRMR < 0.08 (Kline, 2016). Age, gender, and socioeconomic status (SES) were included as covariates in all models.

## 3. Results

### 3.1. Descriptive Statistics and Correlations

Table 1 presents the means, standard deviations, and correlations of the study variables. Social rejection at T1 was positively correlated with anxiety and depression at both T1 and T2. Anxiety and depression at T2 were positively associated with NSSI at T3. By contrast, the association between T1 social rejection and T3 NSSI was weak and statistically non-significant. Furthermore, T2 self-esteem showed a negative correlation with concurrent anxiety and depression (T2).

### 3.2. Mediation Analysis

**Figure 1:**
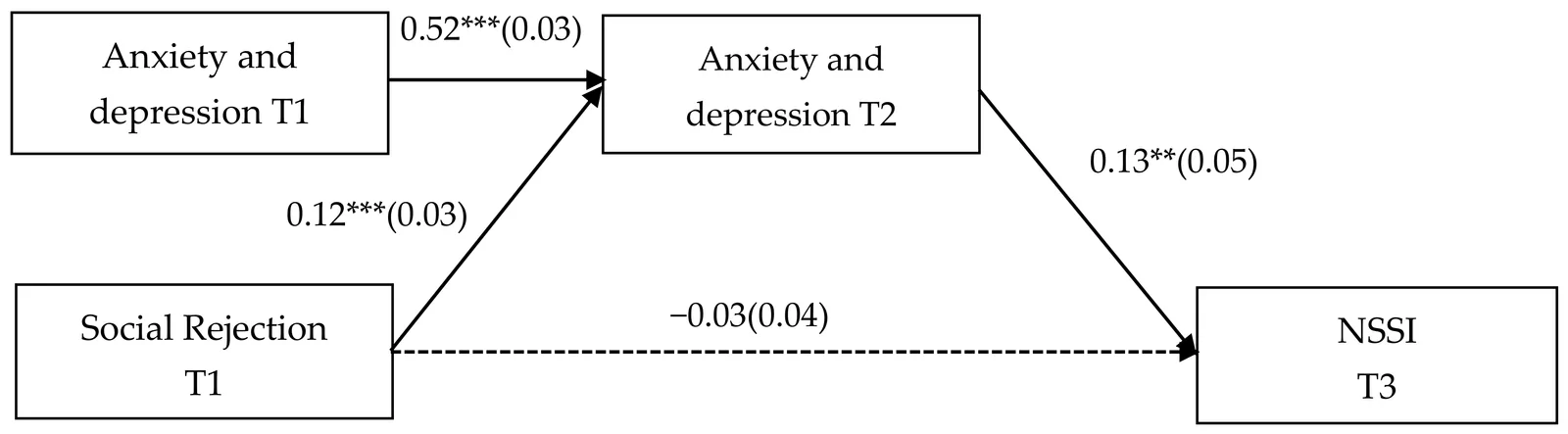
The mediation of T2 anxiety and depression. *Note.* All values represent standardized regression coefficients. Paths involving control variables (e.g., age, gender, SES) are omitted for clarity. Statistically non-significant pathways are denoted by dashed lines. ** *p* < 0.01, *** *p* < 0.001.

Figure 1 summarizes the mediation model results. The model demonstrated acceptable fit: χ^2^(1) = 1.86, χ^2^/df = 1.86, CFI = 0.99, RMSEA = 0.03, SRMR = 0.004. The model explained a significant proportion of the variance in T2 anxiety and depression (*R*^2^ = 0.36, *p* < 0.001) and a small but statistically significant proportion of the variance in T3 NSSI (*R*^2^ = 0.06, *p* < 0.01). After controlling for gender, age, SES, T1 anxiety and depression, and T2 NSSI, higher T1 social rejection predicted increased anxiety and depression at T2 (*β* = 0.12, *SE* = 0.03, *p* < 0.001). Concurrently, T2 anxiety and depression predicted increased T3 NSSI (*β* = 0.13, *SE* = 0.05, *p* < 0.01). Bootstrapping confirmed a significant indirect pathway for T1 social rejection → T2 anxiety/depression → T3 NSSI, indirect effect (IE) = 0.015, *SE* = 0.007, 95% CI [0.002, 0.036]).

### 3.3. Moderated Mediation Analysis

**Figure 2:**
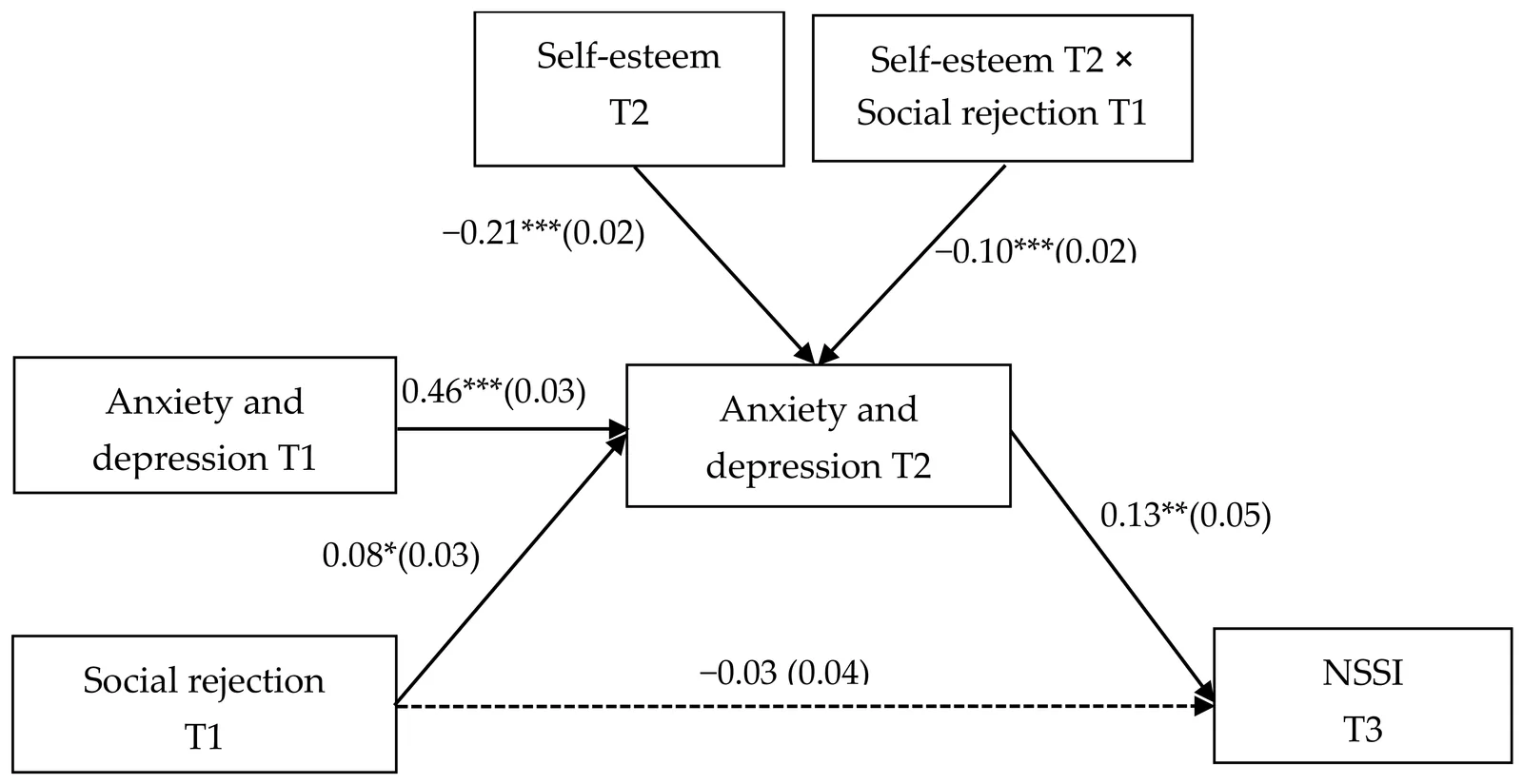
Moderating effect of T2 self-esteem in the mediation effect. *Note*. All values represent standardized regression coefficients. Paths involving control variables (e.g., age, gender, SES) are omitted for clarity. Statistically non-significant pathways are denoted by dashed lines. * *p* < 0.05, ** *p* < 0.01, *** *p* < 0.001.

**Figure 3:**
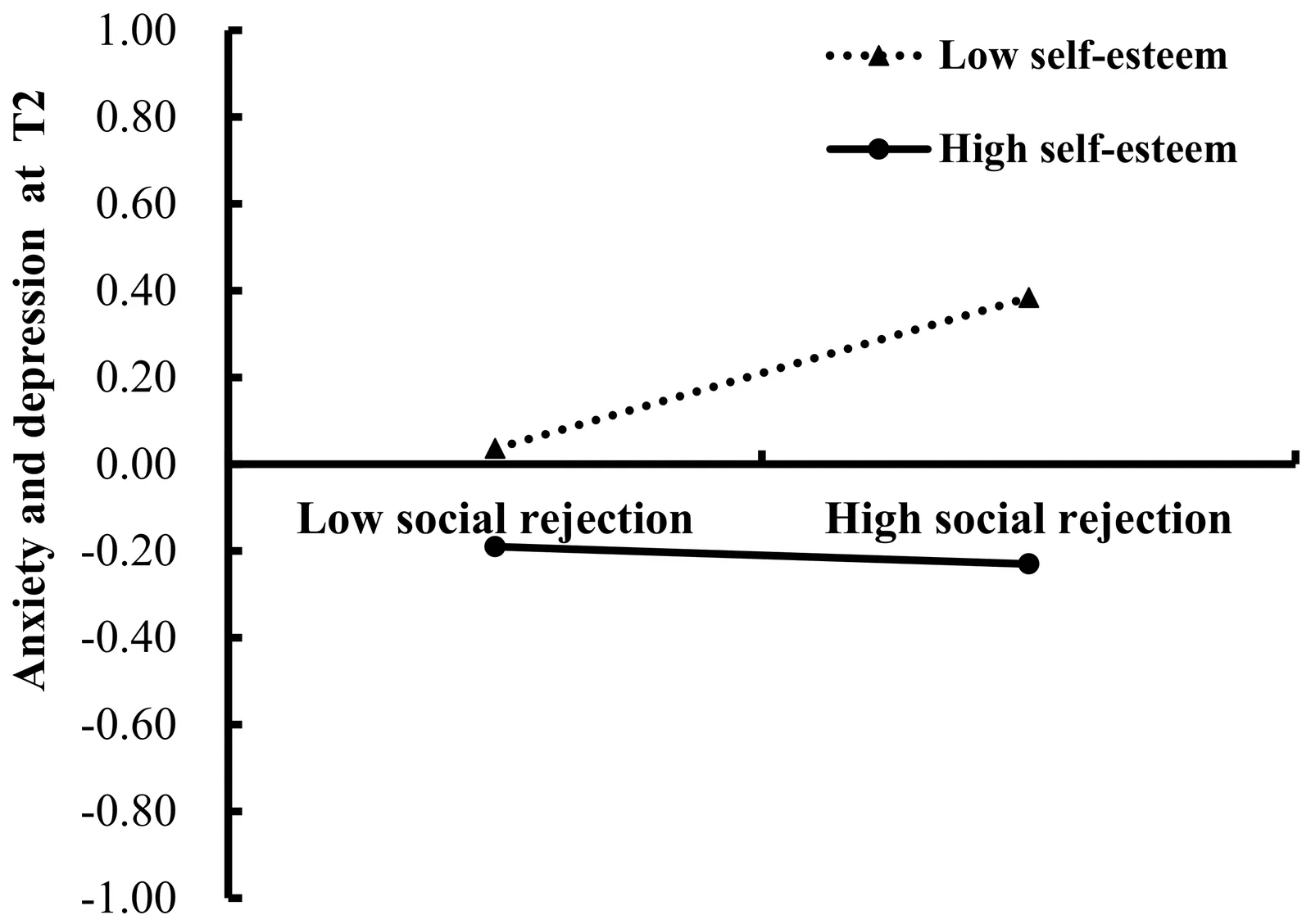
Interactive effect of T1 social rejection and T2 self-esteem on T2 anxiety and depression.

The moderated mediation model demonstrated good fit (Figure 2): χ^2^(3) = 6.39, χ^2^/df = 2.13, CFI = 0.97, RMSEA = 0.03, SRMR = 0.01. Controlling for gender, age, SES, T1 anxiety and depression, and T2 NSSI, T1 social rejection positively predicted T2 anxiety and depression (*β* = 0.08, *SE* = 0.03, *p* < 0.05), T2 anxiety and depression positively predicted T3 NSSI (*β* = 0.13, *SE* = 0.05, *p* < 0.01), and T2 self-esteem was negatively associated with T2 anxiety and depression (*β* = −0.21, *SE* = 0.02, *p* < 0.001). A significant interaction emerged between T1 social rejection and T2 self-esteem on T2 anxiety and depression (*β* = −0.10, *SE* = 0.02, *p* < 0.001). Simple slopes analyses (Figure 3) revealed T1 social rejection significantly predicted T2 anxiety and depression at low self-esteem (1 SD below mean; *β* = 0.17, *SE* = 0.03, *p* < 0.001), but not at high self-esteem (1 SD above mean; *β* = −0.01, *SE* = 0.03, *p* > 0.05). Bias-corrected bootstrapping confirmed a significant conditional indirect effect through T2 anxiety and depression for adolescents with low self-esteem (*β* = 0.03, *SE* = 0.01, 95% CI [0.00, 0.05]), but not high self-esteem (*β* = −0.00, *SE* = 0.01, 95% CI [−0.01, 0.01]). Model comparison further revealed that the interaction term explained an additional 2.0% of the variance in T3 NSSI (Δ*R*^2^ = 0.02), supporting the incremental validity of the moderated mediation model.

## 4. Discussion

This one-year longitudinal study examined the relationship between social rejection and NSSI, along with potential mediating and moderating mechanisms. Results revealed that anxiety and depression serve as mediators in the relationship between social rejection and NSSI. Furthermore, self-esteem moderated this mediating effect. Specifically, individuals with low self-esteem were more likely to experience anxiety and depression following social rejection, which was in turn associated with increased NSSI. In addition, independent variables (social rejection), mediating variables (anxiety and depression), and dependent variables (NSSI) were assessed at three distinct time points to clarify temporal associations (Maxwell & Cole, 2007), a methodology frequently employed in prior research (Li et al., 2023a).

Prior studies have demonstrated that social rejection and self-esteem are independently and strongly associated with NSSI (Cheek et al., 2020; Lin et al., 2023). In this study, a model incorporating social rejection, self-esteem, and NSSI was developed, with a longitudinal follow-up approach used to control for anxiety and depression at T1 and NSSI at T2. The findings revealed that T1 social rejection predicted higher levels of anxiety and depression at T2, which were associated with greater NSSI risk at T3. These findings align with previous research indicating that anxiety and depression mediate the relationship between negative stressful events and NSSI (Li et al., 2023a; Zhu et al., 2021). Additionally, a cross-sectional study has demonstrated that depression mediates the relationship between rejection sensitivity and NSSI (Jiang et al., 2020). To the best of our knowledge, few longitudinal studies have examined the mediating role of anxiety and depression in the relationship between social rejection and self-injury. During adolescence, individuals exhibit heightened attention and concern for social interactions, particularly within the Chinese cultural context, where interpersonal relationships are highly valued (Lou & Li, 2017). Adolescents who experience social rejection often feel anxious or angry about being misunderstood and unaccepted. These defensive cognitive–emotional responses can amplify their behavioral reactions, such as social withdrawal or aggression, leading to increased interpersonal tension (Jiang et al., 2020). Interpersonal tension can be inherently frustrating and painful, conceivably accompanied by feelings of helplessness. This helplessness could be perceived as a lack of personal capability, potentially serving as an internal stressor (e.g., Liu et al., 2014). It is plausible that, over time, this pain and helplessness may contribute to the development or exacerbation of chronic anxiety and depressive symptoms. Subsequently, to alleviate such negative emotional states, adolescents might resort to NSSI as a putative maladaptive emotion regulation strategy. It is important to acknowledge, however, that we did not directly assess the proposed mechanisms, especially helplessness and reinforcement processes. As such, our interpretations are offered as plausible theoretical accounts rather than confirmed empirical pathways, and future work is warranted to test these mechanisms directly.

Notably, the indirect effect of T1 social exclusion on T3 NSSI via T2 anxiety and depression was statistically significant, yet the magnitude of this effect was modest, and thus should be interpreted with due caution. As Adachi and Willoughby (2015) pointed out, when interpreting effect sizes in longitudinal research, one should consider the stability effect, the bivariate correlation, and the degree of attenuation resulting from controlling for prior levels of the outcome. Smaller effect sizes observed in longitudinal models reflect the rigorous standards these designs impose. Therefore, although the effect size observed in this study was smaller than those reported in previous cross-sectional work, such small effects in longitudinal research may still carry substantive significance (Adachi & Willoughby, 2015).

Consistent with our hypothesis, the indirect relationship between social rejection and NSSI through anxiety and depression was moderated by self-esteem. Notably, this moderating effect was significant only among adolescents with low self-esteem. Specifically, adolescents with low self-esteem are more vulnerable to anxiety and depression when facing social rejection, which ultimately increases the risk of NSSI. In contrast, high self-esteem can mitigate the negative effects of social rejection. This finding aligns with previous studies indicating that individuals with high self-esteem possess more coping resources when facing stressful events (Gao et al., 2024). From the perspective of Conservation of Resources (COR) theory (Hobfoll, 1989), this pattern can be understood through the lens of resource loss and resource gain dynamics. More recent developments in COR theory have emphasized the concept of “resource caravans”, the idea that resources do not exist in isolation but tend to travel together, with the presence of one resource facilitating the acquisition and protection of others (Hobfoll et al., 2018). Within this framework, self-esteem can be understood as a key personal resource that, when present, helps individuals maintain other psychological resources (e.g., positive emotions, self-control) and navigate stressful encounters more effectively (Lee et al., 2016). Conversely, when self-esteem is low, individuals experience a deficit in self-control resources (Grodzinsky et al., 2015), making them more vulnerable to resource loss spiral, a process whereby initial resource loss begets further loss (Hobfoll et al., 2018). Applying this framework to the adolescent context, social rejection represents a significant stressor that threatens adolescents’ personal resources (e.g., sense of belonging, social worth). Adolescents with high self-esteem possess greater resource reservoirs to buffer against this threat, enabling them to maintain psychological well-being and avoid maladaptive coping. In contrast, adolescents with low self-esteem lack this resource reserve; when faced with social rejection, they are more prone to resource depletion, manifested as anxiety and depression, which in turn increases the likelihood of engaging in NSSI as a maladaptive strategy to regulate overwhelming negative emotions. This interpretation is consistent with empirical work applying COR theory to adolescent populations, which has shown that personal resources such as self-esteem function as adaptive resources in coping with stress (Howard, 2019; Sanecka et al., 2023). This study further underscores the significance of considering the risk-buffering effect of self-esteem in alleviating the negative impact of social rejection.

It is important to acknowledge that self-esteem is not the only factor that may buffer or modulate the associations observed in this study. A range of other protective and vulnerability factors, including social support, resilience, emotion regulation, and rejection sensitivity, should also be considered when interpreting our findings. Future research would benefit from simultaneously examining multiple protective and risk factors to disentangle their unique, shared, and interactive contributions to the developmental pathways linking social rejection and NSSI. At present, we cannot determine whether self-esteem exerts a unique buffering effect independent of these other variables, or whether its apparent protective role is partially confounded by their influence. Accordingly, our findings regarding self-esteem as a moderator should be interpreted with appropriate caution.

The results of this study have important implications for educators and clinicians. First, our findings indicate that anxiety and depression mediate the relationship between social rejection and NSSI. Thus, interventions that effectively address symptoms of anxiety and depression may help some adolescents break this connection. Consistent with this notion, emotion regulation has been recognized as a promising intervention strategy for managing anxiety symptoms and reducing vulnerability to self-injury (Sloan et al., 2017). Second, theoretically, self-esteem appears to be a meaningful factor to consider when addressing NSSI. Prior research has suggested that enhancing self-esteem may reduce the propensity for self-injurious behavior, as individuals with higher self-esteem may be less likely to engage in NSSI-related pain (Hooley & St. Germain, 2014). Preliminary evidence also indicates that cognitive interventions designed to enhance self-esteem could be effective in reducing vulnerability to NSSI (Hawton et al., 2016). Taken together, given the protective role of self-esteem against NSSI and its positive associations with reduced anxiety and depression, targeted interventions focused on strengthening self-esteem may be a worthwhile consideration for adolescents with low self-esteem who experience social rejection. Such interventions might help mitigate the risk of anxiety, depression, and NSSI. However, it is important to emphasize that these recommendations are derived from observational findings and should be tested empirically in future intervention studies before firm conclusions can be drawn.

## 5. Limitations and Future Direction

The study has several limitations. First, all variables were measured using self-reported methods, which may be influenced by social desirability bias. Using self-reports to obtain information on anxiety, depression, and self-injury is beneficial, as emotional issues are often not easily observed and self-injurious behavior is relatively concealed. Future research would benefit from utilizing multi-method assessments (e.g., interviews and observational data) and incorporating multi-observer reports (e.g., parent and peer evaluations). Secondly, this study only assessed the frequency of seven types of self-injurious behaviors, limiting the depth of information available on self-injury, including crucial aspects such as severity and underlying motivations. Future studies could employ ecological momentary assessment methods, such as daily diary recording, to evaluate both the frequency and severity of participants’ self-injury, which would be more robust (Nock, 2010). Additionally, the absence of T1 NSSI assessment precluded controlling for baseline NSSI. Future research should incorporate baseline measures to more rigorously establish the temporal order of the proposed pathways. Thirdly, this study focused solely on adolescents from one city as the research sample. Future research should aim to broaden the sample size and diversity of participants, investigating whether the present findings can be generalized to rural areas and other age cohorts. Fourthly, the three-wave longitudinal design employed in this study, while improving upon cross-sectional approaches by establishing temporal precedence, does not permit strong causal inferences. Unmeasured third variables may still account for the observed associations, and causal conclusions should therefore be drawn with caution. Future research could extend these findings by applying the Random Intercept Cross-Lagged Panel Model (RI-CLPM), which separates stable between-person variance from within-person change. This would provide a more rigorous test of whether intra-individual fluctuations in social exclusion predict subsequent changes in anxiety, depression, and NSSI. Additionally, the NSSI scale assessed behaviors over the “past 12 months,” while the interval between waves was only 6 months, meaning that the T3 recall window inherently overlapped with the period preceding T2. Although we controlled for prior NSSI to predict residual changes, this overlapping time frame limits our ability to assert that T2 variables exclusively predicted subsequent NSSI occurrences. Future research should employ NSSI measures with recall periods strictly aligned with the intervals between data collection waves, or utilize real-time assessment methods. Fifth, anxiety and depression were combined into a single composite construct in the present study. This decision was theoretically grounded in their high conceptual and empirical overlap, particularly in adolescent populations where they frequently co-occur. It may have obscured potentially distinct mediating roles of anxiety versus depression. Future research with larger samples could examine these two constructs separately to determine whether they operate through differential pathways in linking social rejection to NSSI. Sixth, self-esteem and anxiety/depression were assessed concurrently at T2, which restricts the temporal interpretability of the proposed moderation model. While our theoretical framework posits that self-esteem moderates the prospective relationship between social rejection and subsequent internalizing symptoms, the simultaneous measurement of the moderator and the mediator at T2 precludes conclusions about whether self-esteem moderates the longitudinal (rather than concurrent) association. Future research that assesses self-esteem at different time points will be able to more rigorously examine the moderating effect of self-esteem over time. Seventh, it is worth noting that the direct longitudinal link between T1 social rejection and T3 NSSI was non-significant. This weak direct effect may imply that the temporal interval employed in this study was too long to detect direct fluctuations. Finally, our discussion primarily focused on self-esteem as the protective factor, without directly considering other relevant variables such as social support, resilience, emotion regulation, and rejection sensitivity. This interpretive lens may have oversimplified the complex multifactorial nature of the associations examined. We therefore caution that the role of self-esteem should be understood within a broader constellation of protective and vulnerability processes, and future studies are encouraged to incorporate these additional measures to establish their unique and shared contributions. Furthermore, social rejection was assessed using only four YSR-adapted items. Although validated, this brief measure may not fully capture the construct’s breadth nor adequately distinguish social rejection from related constructs (e.g., peer exclusion, bullying, isolation, rejection sensitivity). Future research using multi-dimensional measures is warranted.

## 6. Conclusions

This three-wave longitudinal study revealed that social rejection is associated with adolescent NSSI through heightened anxiety and depressive symptoms, and that self-esteem moderates this mediating pathway. Specifically, the indirect effect was significant only for adolescents with low self-esteem, whereas it was not significant for those with high self-esteem. This study advances our understanding of social rejection and adolescent NSSI, highlighting the critical roles of emotion regulation and self-esteem enhancement in prevention and intervention.

## Figures and Tables

**Table 1:** Descriptive statistics and correlations.

	1	2	3	4	5	6	7	8	9
1. Gender	1								
2. Age	−0.00	1							
3. SES	−0.06 *	0.14 **	1						
4. T1 Social rejection	0.05 *	0.03	0.11 **	1					
5. T1 AD	0.13 **	−0.01	0.11 **	0.63 **	1				
6. T2 AD	0.08 **	0.02	0.05	0.45 **	0.60 **	1			
7. T2 Self-esteem	−0.01	−0.02	−0.09 **	−0.35 **	−0.37 **	−0.39 **	1		
8. T2 NSSI	−0.11 **	0.00	0.05 *	0.08 **	0.11 **	0.21 **	−0.13 **	1	
9. T3 NSSI	−0.07 **	0.10 **	−0.01	0.03	0.04	0.14 **	−0.09 **	0.20 **	1
*Mean*	-	16.05	3.11	2.08	1.44	1.39	2.63	1.25	1.16
*SD*	-	0.85	0.52	0.98	0.44	0.43	0.38	0.79	0.65

*Note*. *N* = 1368. AD = anxiety and depression, NSSI = non-suicidal self-injury. * *p* < 0.05. ** *p* < 0.01.

## Data Availability

The data supporting this study’s findings are available from the corresponding author upon reasonable request.

## References

[B1-behavsci-16-01281] Achenbach T. M. (1991). Manual for the youth self-report and 1991 profile.

[B2-behavsci-16-01281] Adachi P., Willoughby T. (2015). Interpreting effect sizes when controlling for stability effects in longitudinal autoregressive models: Implications for psychological science. European Journal of Developmental Psychology.

[B3-behavsci-16-01281] Bierman K. L. (2004). Peer rejection: Developmental processes and intervention strategies.

[B4-behavsci-16-01281] Cheek S., Reiter-Lavery T., Goldston D. (2020). Social rejection, popularity, peer victimization, and self-injurious thoughts and behaviors among adolescents: A systematic review and meta-analysis. Clinical Psychology Review.

[B5-behavsci-16-01281] Choi J. W., Hong W., Abela J. R. Z., Cohen J. R. (2020). Comorbid depression and anxiety symptoms in Chinese adolescents: Testing the explanatory power of a diathesis-anxiety model. Research on Child and Adolescent Psychopathology.

[B6-behavsci-16-01281] Cieślak K., Golusiński W. (2018). Coping with loss of ability vs. emotional control and self-esteem in women after mastectomy. Reports of Practical Oncology and Radiotherapy.

[B7-behavsci-16-01281] Cipriano A., Cella S., Cotrufo P. (2017). Nonsuicidal self-injury: A systematic review. Frontiers in Psychology.

[B8-behavsci-16-01281] Cummings C. M., Caporino N. E., Kendall P. C. (2014). Comorbidity of anxiety and depression in children and adolescents: 20 years after. Psychological Bulletin.

[B9-behavsci-16-01281] Gao Y., Liang C., Liu X., Bai R., Xing S. (2024). Self-esteem buffers the stress sensitizing effect of childhood maltreatment on adolescent nonsuicidal self-injury. Journal of Affective Disorders.

[B10-behavsci-16-01281] Gong T., Ren Y., Wu J., Jiang Y., Hu W., You J. (2019). The associations among self-criticism, hopelessness, rumination, and NSSI in adolescents: A moderated mediation model. Journal of Adolescence.

[B11-behavsci-16-01281] Grodzinsky E., Walter S., Viktorsson L., Carlsson A. K., Jones M. P., Faresjö Å. (2015). More negative self-esteem and inferior coping strategies among patients diagnosed with IBS compared with patients without IBS-a case–control study in primary care. BMC Family Practice.

[B12-behavsci-16-01281] Groschwitz R. C., Plener P. L., Groen G., Bonenberger M., Abler B. (2016). Differential neural processing of social exclusion in adolescents with non-suicidal self-injury: An fMRI study. Psychiatry Research: Neuroimaging.

[B13-behavsci-16-01281] Hale W. W., Raaijmakers Q. A. W., Muris P., Van Hoof A., Meeus W. H. J. (2009). One factor or two parallel processes? Comorbidity and development of adolescent anxiety and depressive disorder symptoms. Journal of Child Psychology and Psychiatry.

[B14-behavsci-16-01281] Hawton K., Witt K. G., Salisbury T. L. T., Arensman E., Gunnell D., Hazell P., Townsend E., van Heeringen K. (2016). Psychosocial interventions following self-harm in adults: A systematic review and meta-analysis. Lancet Psychiatry.

[B15-behavsci-16-01281] Heimpel S. A., Wood J. V., Marshall M. A., Brown J. D. (2002). Do people with low self-esteem really want to feel better? Self-esteem differences in motivation to repair negative moods. Journal of Personality and Social Psychology.

[B16-behavsci-16-01281] Hobfoll S. E. (1989). Conservation of resources: A new attempt at conceptualizing stress. American Psychologist.

[B17-behavsci-16-01281] Hobfoll S. E., Halbesleben J., Neveu J. P., Westman M. (2018). Conservation of resources in the organizational context: The reality of resources and their consequences. Annual Review of Organizational Psychology and Organizational Behavior.

[B18-behavsci-16-01281] Hooley J. M., St. Germain S. A. (2014). Nonsuicidal self-injury, pain, and self-criticism: Does changing self-worth change pain endurance in people who engage in self-injury?. Clinical Psychological Science.

[B19-behavsci-16-01281] Howard M. C. (2019). The measurement, nomological net, and theory of perceived self-esteem instability: Applying the conservation of resources theory to understand the construct. Psychological Reports.

[B20-behavsci-16-01281] Jiang Y., Ren Y., Liu T., You J. (2020). Rejection sensitivity and adolescent non-suicidal self-injury: Mediation through depressive symptoms and moderation by fear of self-compassion. Psychology and Psychotherapy-theory Research and Practice.

[B21-behavsci-16-01281] Kiekens G., Hasking P., Boyes M., Claes L., Mortier P., Auerbach R. P., Myin Germeys I., Myin Germeys I. (2018). The associations between non-suicidal self- injury and first onset suicidal thoughts and behaviors. Journal of Affective Disorders.

[B22-behavsci-16-01281] Kline R. B. (2016). Principles and practice of structural equation modeling.

[B23-behavsci-16-01281] Klonsky E. D. (2007). The functions of deliberate self-injury: A review of the evidence. Clinical Psychology Review.

[B24-behavsci-16-01281] Leary M. R., Baumeister R. F. (2000). The nature and function of self-esteem: Sociometer theory. Advances in experimental social psychology.

[B25-behavsci-16-01281] Lee S., Park J., Lee S. B. (2016). The interplay of Internet addiction and compulsive shopping behaviors. Social Behavior and Personality: International Journal.

[B26-behavsci-16-01281] Li Y., Lin S., Han Y., Sheng J., Wang L., Yang X., Chen J. (2023a). Cybervictimization and nonsuicidal self-injury: The mediating role of depressive symptoms and the moderating role of emotional reactivity. Journal of Adolescence.

[B27-behavsci-16-01281] Li Y., Lin S., Yang X., Sheng J., Wang L., Han Y., Cao Y., Chen J. (2023b). A vicious cycle: The reciprocal longitudinal relationship between social rejection, social avoidance, and smartphone addiction among adolescents. International Journal of Mental Health and Addiction.

[B28-behavsci-16-01281] Lin S., Li Y., Sheng J., Chen L., Zhang Y., Chen J. (2025). Non-suicidal self-injury in Chinese adolescents with problematic smartphone use: A one-year longitudinal study. Children and Youth Services Review.

[B29-behavsci-16-01281] Lin S., Li Y., Sheng J., Wang L., Han Y., Yang X., Yu C., Chen J. (2023). Cybervictimization and non-suicidal self-injury among Chinese adolescents: A longitudinal moderated mediation model. Journal of Affective Disorders.

[B30-behavsci-16-01281] Liu R. T., Kraines M. A., Massing-Schaffer M., Alloy L. B. (2014). Rejection sensitivity and depression: Mediation by stress generation. Psychiatry.

[B31-behavsci-16-01281] Lou N. M., Li L. M. W. (2017). Interpersonal relationship mindsets and rejection sensitivity across cultures: The role of relational mobility. Personality and Individual Differences.

[B32-behavsci-16-01281] Marshall S. K., Tilton-Weaver L. C., Stattin H. (2013). Non-suicidal self-injury and depressive symptoms during middle adolescence: A longitudinal analysis. Journal of Youth and Adolescence.

[B33-behavsci-16-01281] Masten C. L., Eisenberger N. I., Borofsky L. A., Pfeifer J. H., Mcnealy K., Mazziotta J. C., Dapretto M. (2009). Neural correlates of social exclusion during adolescence: Understanding the distress of peer rejection. Social Cognitive and Affective Neuroscience.

[B34-behavsci-16-01281] Maxwell S. E., Cole D. A. (2007). Bias in cross-sectional analyses of longitudinal mediation. Psychological Methods.

[B35-behavsci-16-01281] Nock M. K. (2010). Self-injury. Annual Review of Clinical Psychology.

[B36-behavsci-16-01281] Nock M. K., Prinstein M. J. (2004). A functional approach to the assessment of self-mutilative behavior. Journal of Consulting and Clinical Psychology.

[B37-behavsci-16-01281] Ophir Y. (2017). SOS on SNS: Adolescent distress on social network sites. Computers in Human Behavior.

[B38-behavsci-16-01281] Ophir Y., Asterhan C. S., Schwarz B. B. (2019). The digital footprints of adolescent depression, social rejection and victimization of bullying on Facebook. Computers in Human Behavior.

[B39-behavsci-16-01281] Orth U., Robins R. W. (2013). Understanding the link between low self-esteem and depression. Current Directions in Psychological Science.

[B40-behavsci-16-01281] Plener P. L., Schumacher T. S., Munz L. M., Groschwitz R. C. (2015). The longitudinal course of non-suicidal self-injury and deliberate self-harm: A systematic review of the literature. Borderline Personality Disorder and Emotion Dysregulation.

[B41-behavsci-16-01281] Rosenberg M. (1965). Society and the adolescent self-image.

[B42-behavsci-16-01281] Sanecka E., Stasiła-Sieradzka M., Turska E. (2023). The role of core self-evaluations and ego-resiliency in predicting resource losses and gains in the face of the COVID-19 crisis: The perspective of conservation of resources theory. International Journal of Occupational Medicine and Environmental Health.

[B43-behavsci-16-01281] Sloan E., Hall K., Moulding R., Bryce S., Mildred H., Staiger P. K. (2017). Emotion regulation as a transdiagnostic treatment construct across anxiety, depression, substance, eating and borderline personality disorders: A systematic review. Clinical Psychology Review.

[B44-behavsci-16-01281] Valencia-Agudo F., Burcher G. C., Ezpeleta L., Kramer T. (2018). Nonsuicidal self-injury in community adolescents: A systematic review of prospective predictors, mediators and moderators. Journal of Adolescence.

[B45-behavsci-16-01281] Varghese M. E., Pistole M. C. (2017). College student cyberbullying: Self-esteem, depression, loneliness, and attachment. Journal of College Counseling.

[B46-behavsci-16-01281] West K. (2018). Naked and unashamed: Investigations and applications of the effects of naturist activities on body image, self-esteem, and life satisfaction. Journal of Happiness Studies.

[B47-behavsci-16-01281] Westenberg P. M., Gullone E., Bokhorst C. L., Heyne D. A., King N. J. (2007). Social evaluation fear in childhood and adolescence: Normative developmental course and continuity of individual differences. British Journal of Developmental Psychology.

[B48-behavsci-16-01281] Yu C., Li X., Wang S., Zhang W. (2016). Teacher autonomy support reduces adolescent anxiety and depression: An 18-month longitudinal study. Journal of Adolescence.

[B49-behavsci-16-01281] Zheng Y., Yang X., Liu Q., Chu X., Huang Q., Zhou Z. (2020). Perceived stress and online compulsive buying among women: A moderated mediation model. Computers in Human Behavior.

[B50-behavsci-16-01281] Zhou J., Gong X. (2023). Longitudinal relation between maladaptive parenting and nonsuicidal self-injury among Chinese early adolescents: The roles of internalizing symptoms and FKBP5 gene variation. Journal of Affective Disorders.

[B51-behavsci-16-01281] Zhou J., Li X., Tian L., Huebner E. S. (2020). Longitudinal association between low self-esteem and depression in early adolescents: The role of rejection sensitivity and loneliness. Psychology and Psychotherapy: Theory, Research and Practice.

[B52-behavsci-16-01281] Zhu J., Chen Y., Su B., Zhang W. (2021). Anxiety symptoms mediates the influence of cybervictimization on adolescent non-suicidal self-injury: The moderating effect of self-control. Journal of Affective Disorders.

[B53-behavsci-16-01281] Zou H., Tang D., Chen Z., Wang E. C., Zhang W. (2024). The effect of social anxiety, impulsiveness, self-esteem on non-suicidal self-injury among college students: A conditional process model. International Journal of Psychology.

